# Pulmonary Hyalinizing Granuloma Presenting as Diffuse Nodular Lung Disease in a Stone Worker: A Case Report

**DOI:** 10.1155/crpu/5061557

**Published:** 2026-11-10

**Authors:** Hanien Samara, Henrik Ghantarchyan, Sarkis Arabian

**Affiliations:** ^1^ Osteopathic Medicine College of Osteopathic Medicine of the Pacific Western University of Health Sciences Pomona California USA westernu.edu; ^2^ Pulmonary Critical Care Arrowhead Regional Medical Center Colton California USA arrowheadmedcenter.org

**Keywords:** case report, pulmonary hyalinizing granuloma

## Abstract

Pulmonary hyalinizing granuloma (PHG) is a rare pseudoneoplastic condition characterized by the development of benign, often multifocal nodular lesions. Because patients may have few or no respiratory symptoms, the condition is frequently identified incidentally during imaging performed for unrelated indications. Radiographically, PHG can manifest as numerous bilateral pulmonary nodules accompanied by mediastinal or hilar lymphadenopathy. Histopathologic examination typically demonstrates dense, acellular hyalinized material arranged in lamellar patterns with surrounding perivascular inflammatory changes. Although the pathogenesis remains incompletely defined, proposed mechanisms include abnormal immune responses triggered by infectious or autoimmune processes. We report the case of a 49‐year‐old man with substantial occupational exposure to stone and tile materials and a history of social inhalational exposures. He immigrated from Mexico in the early 2000s and subsequently worked for many years in tile and stone installation, reportedly with consistent use of personal protective equipment. He initially presented to the emergency department after a workplace accident in which a granite slab fell onto his back. CT imaging obtained during this evaluation unexpectedly demonstrated extensive bilateral pulmonary abnormalities, including hilar and mediastinal lymphadenopathy, a diffuse miliary‐appearing pattern, and multifocal areas of patchy consolidation. Given the unusual radiographic findings, an extensive infectious evaluation, including fungal studies, was performed and did not identify an infectious cause. Cardiothoracic surgery subsequently performed VATS with lung wedge biopsy. Intraoperatively, diffuse miliary‐appearing lesions were observed throughout both lungs along with enlarged, firm, rubbery lymph nodes. Histopathologic examination of the wedge specimen demonstrated findings compatible with pulmonary hyalinizing granuloma and associated anthracosis. During subsequent outpatient pulmonary evaluation, PFTs demonstrated severe obstructive physiology without a significant bronchodilator response. Repeat CT imaging 3 months later showed worsening nodular changes, and the patient was ultimately referred for lung transplantation evaluation. The extensive occupational history raised consideration of pneumoconiosis, particularly silica‐related disease, given his longstanding work with stone materials. However, the combination of the radiographic pattern, histopathologic findings, negative infectious evaluation, and reported use of appropriate respiratory protection supported PHG as the primary diagnosis. Although the relationship between PHG and occupational exposures remains uncertain, this case highlights the diagnostic challenge posed by diffuse nodular lung disease in patients with significant environmental exposure histories. Further investigation into the inflammatory and immune‐mediated mechanisms underlying PHG may help clarify its relationship with infectious, autoimmune, and environmental factors.

## 1. Introduction

Pulmonary hyalinizing granuloma (PHG) is a rare benign fibrosing lung disease that often presents as solitary or multiple pulmonary nodules. Findings are often incidentally noted on imaging, with mild or asymptomatic presentation. Chest imaging traditionally exhibits widely disseminated pulmonary nodules and mediastinal adenopathy. Histological analysis of such nodules reveals homogeneous hyaline lamellae with perivascular cuffing [1, 2]. This case report focuses on a case of PHG in a 49‐year‐old male with significant occupational and inhalational exposures to help us better understand the disease process and clinical presentation.

This article was previously presented as a meeting abstract at the 2026 American Thoracic Society (ATS) Annual Scientific Meeting on May 17–18, 2026.

## 2. Case Presentation

The patient is a 49‐year‐old male with a history of tobacco use (one to two cigarettes a month, quit 20 years ago), intermittent cocaine use, and inhalant illicit drug use, who initially presented to the ED for left upper extremity and head pain for trauma status post granite falling onto his back while at work. He emigrated from Mexico in the early 2000s and has been employed in the tile and stone installation business for the majority of his adult life. The patient endorsed appropriate personal protective equipment (PPE) use. The patient reported associated intermittent cough with wheezing, fatigue, weight loss (20 pounds within the last year), myalgias, and shortness of breath when ambulating longer distances than usual; no fevers, chills, hemoptysis, night sweats, or abdominal pain.

Chest X‐ray was notable for bilateral bronchovascular markings and diffuse nodular opacities (Figure 1). Computed tomography (CT) of the chest revealed findings of moderate left pneumothorax, diffuse miliary pattern, patchy consolidations throughout bilateral lung fields, and mediastinal and hilar lymphadenopathy (Figure 2). The left pneumothorax was clinically considered traumatic in the setting of the patient′s recent blunt chest trauma and was managed with placement of a left thoravent, which was subsequently removed the following day. Labs pertinent for mild leukocytosis (white blood cell count of 13.7). Fungal and infectious studies were negative for legionella antigen, urine lipoarabinomannan, *Streptococcus pneumoniae* urine antigen, *Coccidioides* antibody, mycoplasma pneumonia antibody, mycelial phase antibody (histoplasma), yeast phase antibody (histoplasma), and acid‐fast bacilli (AFB) smear X3 and fungal/anaerobic culture.

**Figure 1:**
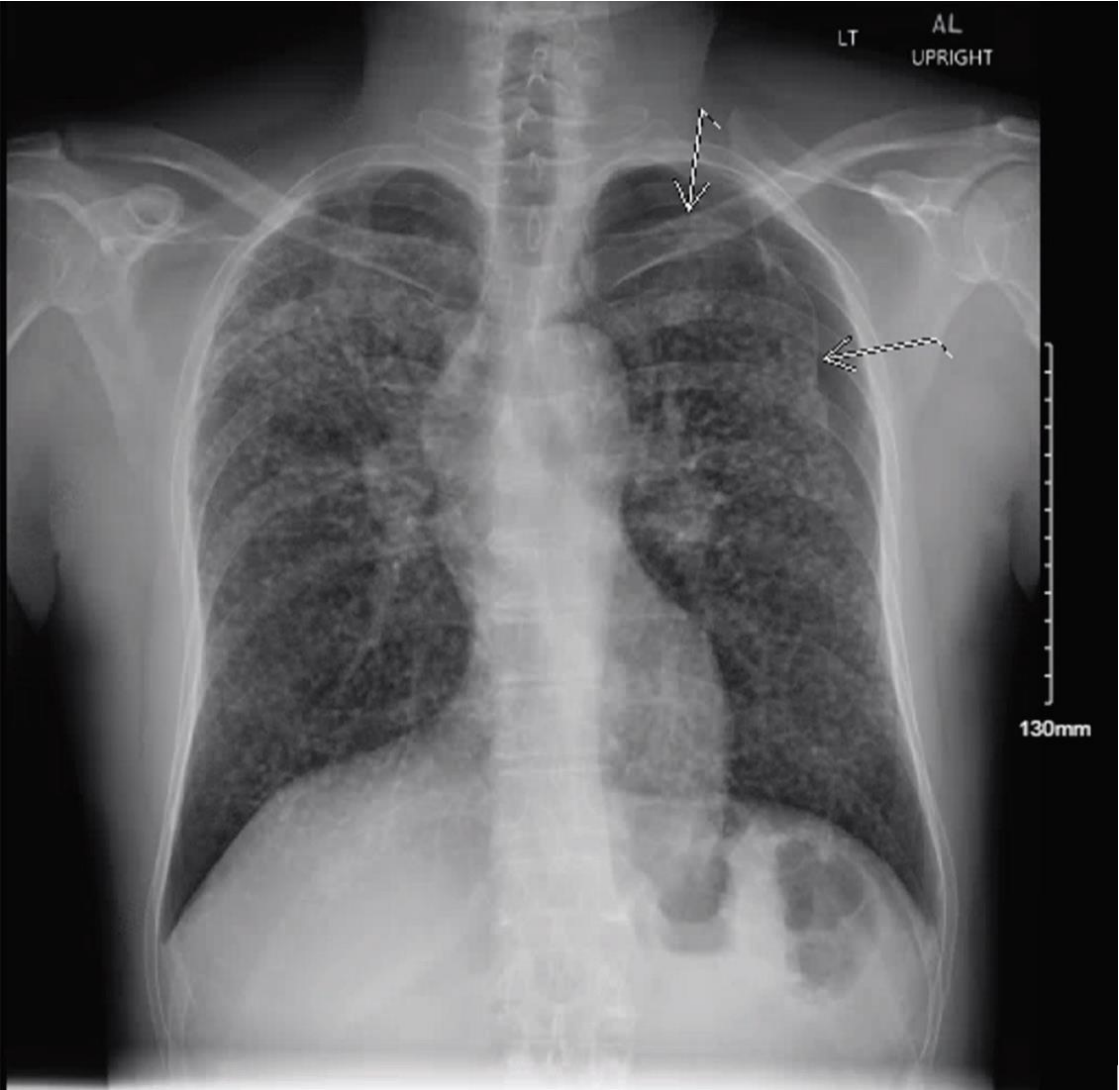
Chest X‐ray notable for bilateral bronchovascular markings and diffuse nodular opacities.

**Figure 2:**
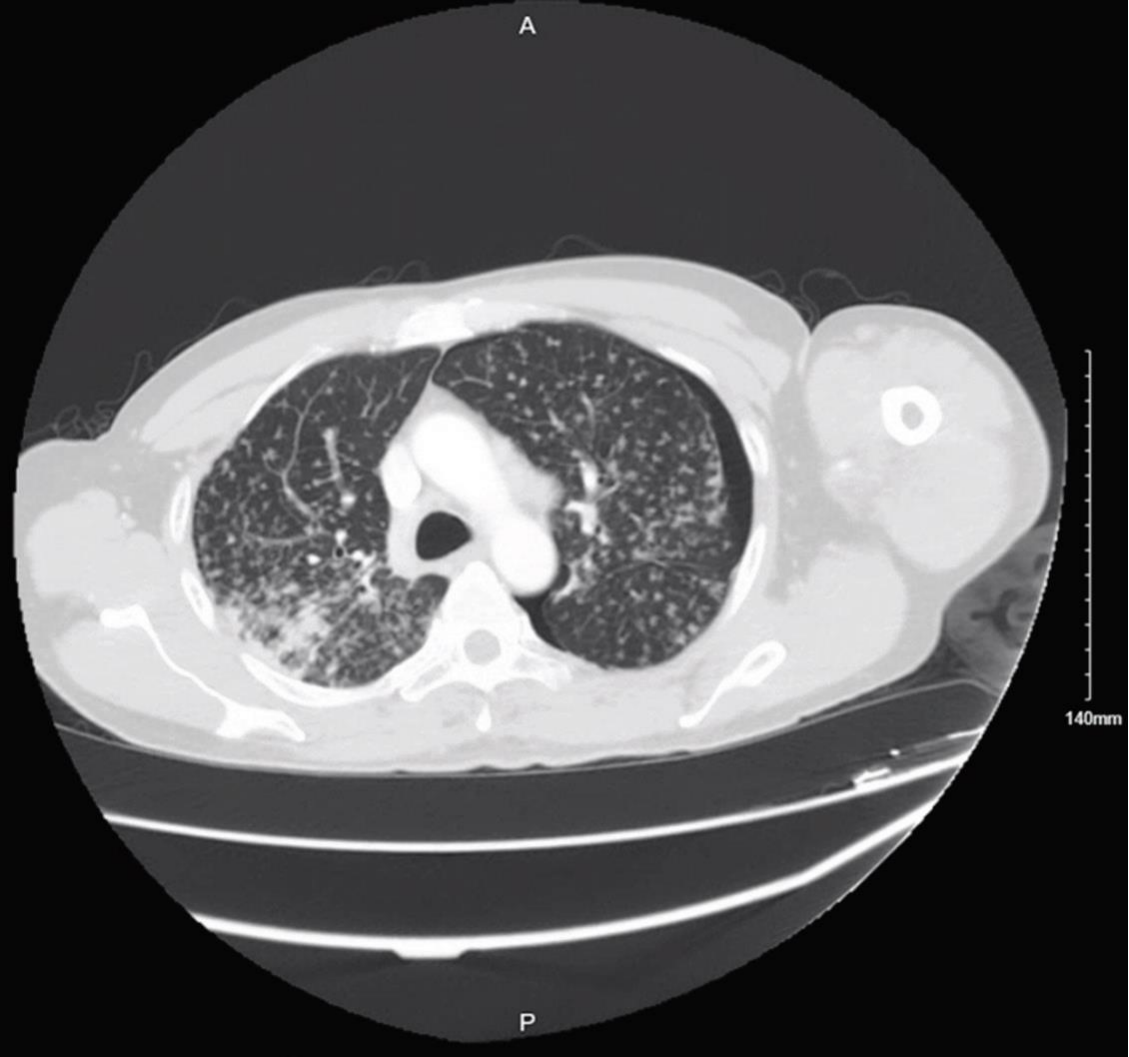
CT chest showing diffuse miliary nodularity, patchy areas of consolidation, and left pneumothorax.

Cardiothoracic surgery was consulted and the patient underwent video‐assisted thoracic surgery (VATS). Miliary pattern was found throughout both lungs and large rubber‐textured lymph nodes. Wedge biopsy was positive for hyalinizing granuloma and anthracosis. Pulmonary function testing (PFTs) showed evidence of severe obstruction with no response to bronchodilators, mild restriction, air trapping, and mild diffusion impairment (Figure 3).

**Figure 3:**
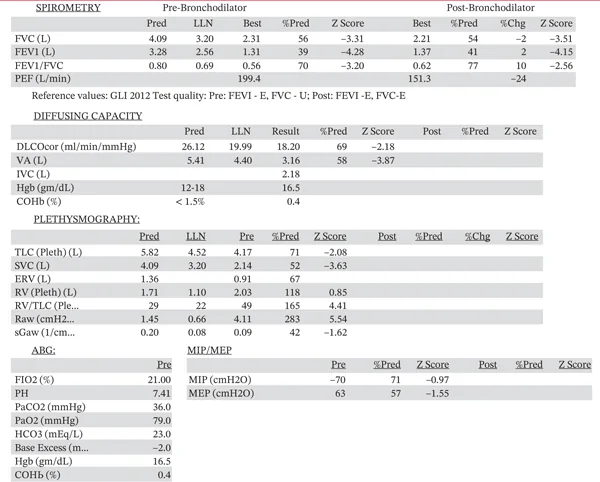
Pulmonary function testing (PFTs) showing severe obstructive and mild restrictive pattern without improvement with bronchodilators.

In the outpatient setting, the patient was started on albuterol (ventolin HFA) 90 mcg/actuation inhaler as needed for intermittent cough with mild wheezing. Repeat CT chest 3 months later showed worsening nodular changes (Figure 4). The patient was ultimately recommended for lung transplantation evaluation and continues to follow outpatient with pulmonology.

**Figure 4:**
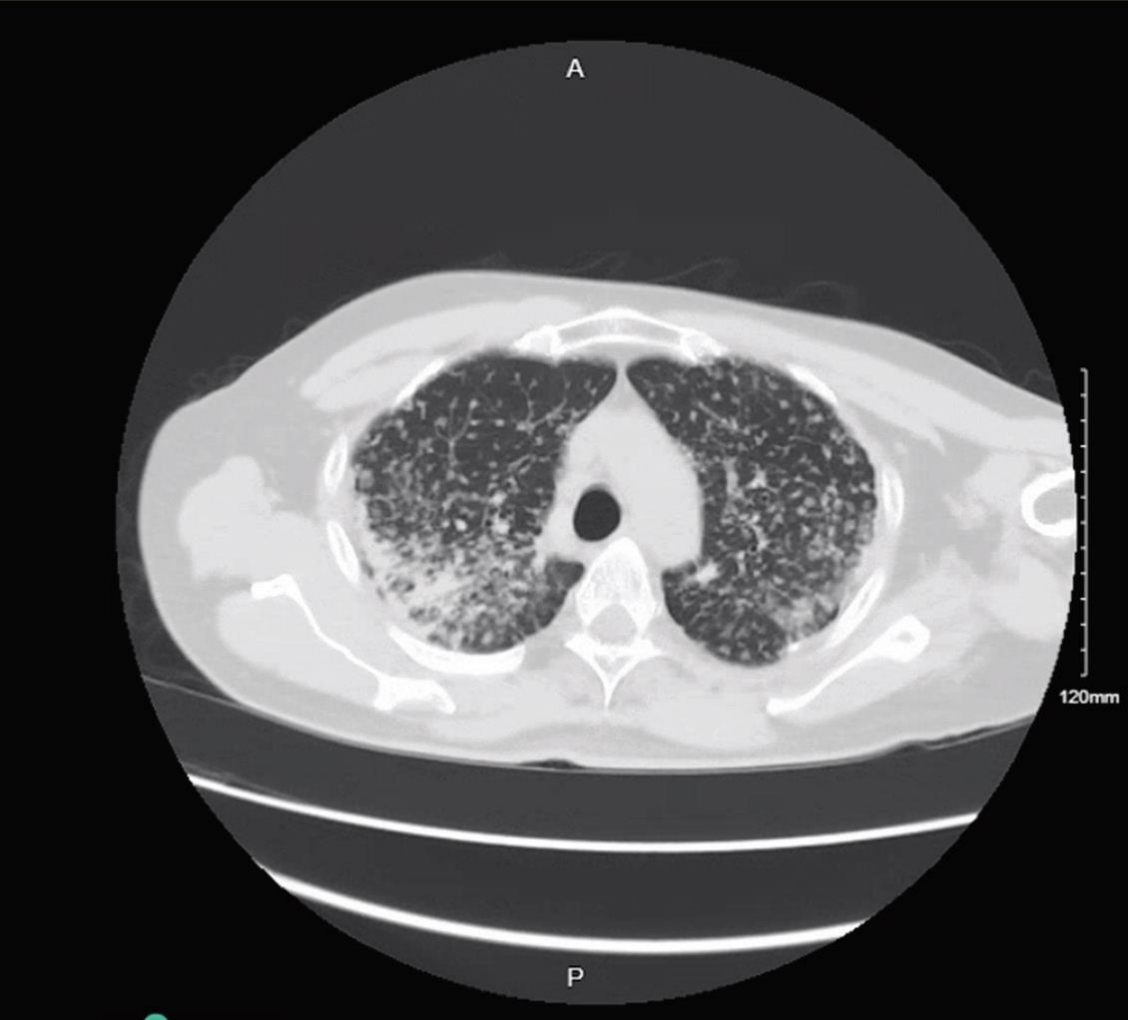
Repeat CT chest 3 months after initial encounter displaying diffuse subcentimeter nodular lesions in bilateral upper lobes.

## 3. Discussion

The discovery of PHG is often an incidental finding on imaging with conclusive diagnosis via biopsy. Initially detailed by Benfield et al. in 1962, patients are often asymptomatic or exhibit mild respiratory symptoms at presentation [1]. The presence of well‐defined, randomly distributed pulmonary nodules of varying sizes on imaging (ranging from millimeters to several centimeters in diameter) and the presence of mediastinal and hilar adenopathy often mimics metastatic disease [2]. Approximately 60% of PHG nodules display increased metabolic activity using fluorodeoxyglucose (FDG) positron emission tomography (PET) (FDG‐PET) [3]; hence, a biopsy (typically via VATS procedure) is required for formal diagnosis. Current literature suggests that transbronchial cryobiopsy may offer a less invasive alternative for diagnostic testing compared with standard wedge biopsy, although additional evaluation on this matter is still necessary [4]. Although the underlying mechanism of PHG is still unclear, previous case reports have displayed an association with autoimmune and infectious processes. Specifically, Lhote et al. found that out of 135 cases of PHG cited in literature, 19 were correlated with infectious etiology (most commonly tuberculosis) and 17 cases were associated with autoimmune diseases [5]. Additionally, several case reports have displayed an association between elevated serum IgG4 levels and PHG [6]. Histological analysis of PHG nodules reveals homogeneous hyaline lamellae with immune cells (plasma cells, lymphocytes, and histiocytes) surrounding the perivasculature, further reinforcing an underlying role in abnormal immune response [7]. Wedge biopsy demonstrated findings consistent with hyalinizing granuloma and anthracosis. Additional representative pathological images were not available for inclusion in this report. Furthermore, the available pathology documentation did not provide sufficient detail regarding specific features such as calcification, necrosis, granulomatous inflammation, or vascular and perivascular involvement. The presence of anthracosis may reflect chronic exposure to inhaled particulate matter; however, its specific relationship to the patient′s occupational exposure, remote tobacco use, or intermittent inhalational drug use cannot be determined. Although the patient had a longstanding occupational history involving marble and stone cutting, a direct causal relationship between this exposure and PHG cannot be established. Occupational particulate exposure may represent a potential associated factor in this case, particularly given the accompanying anthracosis, but further studies are needed to determine whether such exposures contribute to the development of PHG. The patient′s history of intermittent cocaine and inhalational illicit drug use represents another potential contributor to his pulmonary findings. Inhalational exposures may cause airway and parenchymal injury and could potentially contribute to the severe obstructive physiology demonstrated on PFTs.

The extensive infectious evaluation was unrevealing, including testing for bacterial, fungal, mycobacterial, and atypical infectious etiologies. Given the reported associations between PHG and autoimmune or IgG4‐related disease, evaluation for systemic autoimmune disease and serum IgG4 may also be considered when clinically appropriate. In this case, the absence of a documented autoimmune or IgG4 evaluation acts as a limitation. Although corticosteroid therapy has been reported in selected cases [5, 6, 8], its role in PHG remains uncertain, and our patient was not treated with corticosteroids before repeat imaging. Referral for lung transplantation evaluation reflected the severity and progression of the patient′s underlying pulmonary disease despite the absence of a definitive medical therapy.

Another key differential diagnosis considered for this patient was silicosis. Traditionally, pneumoconioses (such as silicosis) occur following prolonged occupational exposure to particulate matter. Common examples include coal mining, construction, and stone fabrication [9, 10]. Histologically, silicosis is classified by a silicotic nodule, consisting of a central, hyalinized, and whorled collagen surrounded by fibrosis. More specifically, under polarized light microscopy (PLM), silica manifests as a birefringent crystalline particle [11]. On chest imaging, chronic silicosis typically presents with upper‐lobe‐predominant subcentimeter nodules and hilar adenopathy, whereas PHG more commonly demonstrates multiple, well‐defined nodules that may be more diffusely distributed. Despite the fact that our patient did have significant exposure to stone/marble cutting, PLM and mineralogical analysis were not performed; therefore the presence of birefringent silica particles could not be definitively assessed. This represents a limitation of the current case and prevents complete exclusion of concomitant silica‐related lung disease, although overall clinical presentation and imaging are more consistent with PHG.

## 4. Conclusion

PHG is a rare benign pulmonary disease hallmarked by the presence of diffuse nodules on imaging and hyaline lamellar bodies on biopsy. Patients are often minimally symptomatic on presentation, and its manifestation is thought to be linked to infectious and/or autoimmune etiology. This case report denoting a middle‐aged male with various environmental/social exposures further exemplifies a likely inflammatory component in the disease process.

## Funding

No funding was received for this manuscript.

## Consent

Written informed consent was obtained from the patient for publication of this case report and any accompanying images. The patient was informed that identifying information would be omitted to protect privacy.

## Conflicts of Interest

The authors declare no conflicts of interest.

## Data Availability

The data that support the findings of this study are available on request from the corresponding author. The data are not publicly available due to privacy or ethical restrictions.
